# Aluminium – Addendum: re-evaluation of the BAT Value and assignment to a pregnancy risk group: Assessment Values in Biological Material – Translation of the German version from 2025

**DOI:** 10.34865/bb742990e10_3ad

**Published:** 2025-09-29

**Authors:** Sandra Michaelsen, Wobbeke Weistenhöfer, Rüdiger Bartsch, Nadine Hund, Gerlinde Schriever-Schwemmer, Hans Drexler, Andrea Hartwig, MAK Commission

**Affiliations:** 1 Institute of Applied Biosciences. Department of Food Chemistry and Toxicology. Karlsruhe Institute of Technology (KIT) Adenauerring 20a, Building 50.41 76131 Karlsruhe Germany; 2 Friedrich-Alexander-Universität Erlangen-Nürnberg. Institute and Outpatient Clinic of Occupational, Social, and Environmental Medicine Henkestraße 9–11 91054 Erlangen Germany; 3 Permanent Senate Commission for the Investigation of Health Hazards of Chemical Compounds in the Work Area. Deutsche Forschungsgemeinschaft, Kennedyallee 40, 53175 Bonn, Germany. Further information: Permanent Senate Commission for the Investigation of Health Hazards of Chemical Compounds in the Work Area | DFG

**Keywords:** aluminium, biological tolerance value, BAT value, developmental toxicity, developmental neurotoxicity, pregnancy risk group

## Abstract

The German Senate Commission for the Investigation of Health Hazards of Chemical Compounds in the Work Area (MAK Commission) re-evaluated the data for aluminium [7429-90-5] to verify the biological tolerance value (BAT value) of 50 μg aluminium/g creatinine in urine and assign it to a pregnancy risk group. Relevant studies were identified from a literature search. In the previous evaluation, neurotoxic effects were considered the most sensitive systemic endpoint of aluminium and a BAT value of 50 μg aluminium/g creatinine was derived from a no observed adverse effect level (NOAEL) of 50 μg/g creatinine for the occurrence of preclinical neurotoxic effects in humans, which was determined by standardised neuropsychological test procedures in workplace studies. As the BAT value is thus well-founded and there are no more recent data that would call this into question, the BAT value for aluminium is confirmed. Sampling time is at the end of the shift, for long-term exposures after several previous shifts. There are no reliable studies available to assess the developmental toxicity and developmental neuroxicity of aluminium compounds in humans. There is no evidence that children are more sensitive to aluminium-induced neurotoxic effects than adults. In animal studies, aluminium concentrations in the urine of pregnant animals have not been determined and it is not known at which blood concentration developmental toxic or developmental neurotoxic effects occur in animals. There are also uncertainties regarding the transfer of animal data to humans. Therefore, a reliable risk assessment for developmental toxicity and developmental neurotoxicity is not possible and with regard to the BAT value of 50 µg aluminium/g creatinine, aluminium is assigned to Pregnancy Risk Group D.

**Table:** 

**BAT value (2017)**	**50 μg aluminium/g creatinine**
	Sampling time: at the end of the shift, for long-term exposures after several previous shifts
Prenatal toxicity (2024)	Group D
**BAR (2018)**	**15 μg aluminium/g creatinine**
	
**MAK value (2024)**	**–**
**Poorly soluble compounds**	**0.05 mg Al/m^3^ R** **0.5 mg Al/m^3^ I**
Carcinogenicity (2024)	4^a)^
Prenatal toxicity (2024)	Group D
**Soluble compounds**	**0.005 mg Al/m^3^ I for aluminium chlorohydrate** **0.0002 mg Al/m^3^ I for aluminium chloride, aluminium citrate, aluminium lactate, aluminium nitrate, aluminium sulfate**
Prenatal toxicity (2024)	Group C
Absorption through the skin (2024)	–

^a)^ particle overload effect in the lungs

## Re-evaluation

In 2017, neurotoxic effects were considered the most sensitive systemic endpoint of aluminium. A BAT value of 50 μg aluminium/g creatinine was derived based on a NOAEL of 50 μg aluminium/g creatinine for the occurrence of preclinical neurotoxic effects in humans, which was determined by standardised neuropsychological test procedures in workplace studies (translated in Klotz et al. 2019).

In 2024, due to the local lung effects in rats (increased inflammation markers in the BALF (bronchoalveolar lavage fluid), slight hypercellularity and focal septal collagen deposits in the bronchoalveolar region, increased absolute lung weight, increased absolute and relative weights of the lung-associated lymph nodes and particles in the alveoli and alveolar macrophages), a maximum workplace concentration **(MAK value) for poorly soluble aluminium compounds** of 0.05 mg aluminium/m^3^ R (respirable fraction) was set. Due to the irritation of the respiratory tract in animal experiments, **MAK values for soluble aluminium compounds** of 0.005 mg Al/m^3 ^I (inhalable fraction) for aluminium chlorohydrate and 0.0002 mg Al/m^3^ I for aluminium chloride, aluminium citrate, aluminium lactate, aluminium nitrate and aluminium sulfate were derived.

As exposure at the level of the MAK value for the respirable fraction would lead to a concentration in biological material below the BAT value, this value must be re-evaluated. In addition, the BAT value has to be assigned to a pregnancy risk group.

## Toxicokinetics

The bioavailability of aluminium compounds is complex. It depends on the **water solubility**, the pH and hydration of the individual aluminium compound; in the case of inhalation, the **particle size** also plays a role. The route and duration of administration also cause differences in the bioavailability and toxicokinetic behaviour of aluminium. In rodents and humans, there are differences in terms of storage capacity, relation to creatinine and half-lives in organs/tissues (longer in humans compared with rats, see physiologically based pharmacokinetic (PBPK) model for single oral intake; Hethey et al. 2021). PBPK models for longer exposure durations are not available. In addition, the considerable risk of contamination in pre-analysis procedures must be emphasised.

The **oral bioavailability** of aluminium is very low at around 0.1% in humans after ingestion via food but can vary about 10-fold (EFSA 2008. Bioavailability following **inhalation of soluble aluminium compounds **is 5% (data from employees exposed to aluminium at the workplace; Pierre et al. 1995). For** inhaled poorly soluble aluminium compounds, **bioavailability is lower at around 2% (Priest 2004).

High aluminium levels are found particularly in the skeleton. While aluminium is released comparatively quickly from most tissues and excreted via the kidneys, elimination from the bones is very slow, with a half-life of several years. Chronic exposure therefore leads to an** accumulation of aluminium in the bones** (EFSA 2008; Hellström et al. 2005).

At the workplace, an** accumulation of readily available aluminium over the working week** is assumed which is of relevance for the selection of the sampling time.

Data on background levels of aluminium in the general population show **great variability in serum or plasma levels**. According to the Federal Environmental Agency, the reference range for aluminium in serum is < 5 μg/l (HBM-Kommission 1998).

## Epidemiological studies

### Effects on the respiratory system

Studies on respiratory effects after repeated exposure to aluminium at the workplace were described in detail (translated in Hartwig 2013) and summarised in Hartwig and MAK Commission (2025 a) including the results of studies published after 2005.

None of the described studies showing effects on the respiratory system is sufficiently conclusive to derive a limit value in the biological material for lung changes. The study by Letzel et al. (2006) showed for the years 1999 to 2003 at low measured air concentrations in the range of 0.47–0.76 mg aluminium(oxide)-containing welding fumes/m^3^ and medians of 62.45–135.8 µg aluminium/g creatinine in urine and 8.7–15.56 µg aluminium/l in plasma (mean value before/after shift) shows lung effects. However, these are not attributable to aluminium alone, but probably to co-exposure to ozone or an influence by a high rate of smokers or former smokers in the study collective.

In the study by Hałatek et al. (2006) investigating 50 smelters with exposures to Al_2_O_3_ of 0.32 ± 0.18 mg/m^3^ and an average of 43.7 (± 23.7) µg aluminium/l urine, 42 control persons and 16 other employees exposed to aluminium, the lung function parameters of the smelters were not statistically significantly changed in comparison with the control persons. The group with the highest aluminium exposure in the air did not show a statistically significant decrease in club cell protein (CC16) as a sign of chronic exposure. CC16 is a small, anti-inflammatory protein that is secreted almost exclusively by the club cells of the terminal bronchial epithelium. In addition, CC16 is also influenced by other factors such as smoking, ambient temperature, time of day or infections.

Kraus et al. (2006) examined 62 workers in two aluminium powder production plants and found a significant correlation between the development of aluminosis and aluminium concentrations in urine at and above 200 µg/g creatinine using high-resolution computer tomography (HRCT).

### Effects on the central nervous system 

The occurrence of preclinical neurotoxic effects was considered the most sensitive endpoint for deriving the BAT value. These effects were recorded using standardised neuropsychological test procedures in workplace studies, which were described in detail in Klotz et al. (2019). In Hartwig and MAK Commission (2025 a), workplace studies from 2017 onwards, other relevant studies as well as meta-analyses, case reports and patient and environmental studies were presented. At the workplace, exposures occur primarily to poorly soluble aluminium compounds. The available studies showed that the concentration of aluminium in urine did not correlate with the air concentration (Kiesswetter et al. 2007). No epidemiological studies are available for soluble aluminium compounds (Hartwig and MAK Commission 2025 b).

Recent workplace studies from various regions of China showed very high aluminium levels in plasma or serum in both aluminium-exposed and non-exposed control subjects (Meng et al. 2019 a, b; Shang et al. 2020, 2021). This indicates a high background exposure to aluminium in the investigated regions of China or contamination of the samples. The aluminium blood levels in the studies significantly exceeded the reference value of the German general population (< 5 µg/l serum) and were partly in the range in which a poorer performance in neuropsychological tests and neurotoxic effects (> 13 µg/l plasma) were observed (Klotz et al. 2019).

The relationship between plasma aluminium and neurotoxic effects was investigated in 392 male electrolysis workers in China using regression analysis (Zhang et al. 2022). Four groups of 98 workers each were formed based on aluminium levels in plasma (group 1: < 18.08 µg/l plasma (12 ± 8 years of exposure), group 2: 18.08–28.2 µg/l (14 ± 10 years of exposure), group 3: 28.2–40.88 µg/l (16 ± 10 years of exposure) and group 4: > 40.88 µg/l (19 ± 7 years of exposure)). The average age of the employees was 40 ± 7.4 years, and they worked for the company for an average of 15.1 ± 8.8 years. The aluminium dust concentration (probably inhalable fraction) is given as 1.07–2.13 mg/m^3^. Data on age, level of education, marital status, years worked, lifestyle (smoking status, drinking habits) and personal and family medical histories were recorded in a questionnaire, cognitive functions were analysed and blood pressure was determined. Subjects with higher aluminium concentrations in their plasma performed worse in neuropsychological tests compared to the group with the lowest concentrations. This was demonstrated by a negative correlation between aluminium concentration and the results of the Mini-Mental Status Examination (MMSE) (p for trend < 0.05) and the Verbal Fluency Test (VFT) (p for trend < 0.05). Although the Fuld Object Memory Evaluation (FOM) showed a trend in the same direction, it did not reach statistical significance. In contrast, the average reaction time tended to be faster in subjects with higher aluminium concentrations (positive correlation, p for trend < 0.05). Adjustments were made for age, level of education, marital status, smoking, drinking, years worked and body mass index. Only for MMSE and VFT was it possible to model a concentration–response relationship with regard to the aluminium plasma concentration. Furthermore, the results showed that group 4 (> 40.88 µg aluminium/l plasma) compared with group 1 (< 18.08 µg aluminium/l plasma) had increased risks for hypertension (prevalence ratio (PR) = 2.75; 95% CI (confidence interval): 1.24–6.09), increased systolic (PR = 2.6; 95% CI: 1.1–6.1) and diastolic blood pressure (PR = 3.36; 95% CI: 1.29–8.79). The modelling showed that hypertension as well as systolic and diastolic blood pressure influenced the aluminium-induced decrease in MMSE score. Hypertension had the strongest effect (16.3%), followed by systolic blood pressure (14.2%) and diastolic blood pressure (11.2%). Hypertension and diastolic blood pressure also affected the aluminium-induced decrease in VFT score by 9.4% and 10.7%, respectively. The aluminium concentrations in plasma measured in this study are already in a range in which neurotoxic effects are to be expected.

The workplace studies by Deschamps et al. (2009, 2018), which have also been published since the last documentation, were presented in detail in Hartwig and MAK Commission (2025 b) and are not reliable due to methodological shortcomings.

Other workplace studies cannot be used to assess neurotoxicity either, as there was exposure to other metals (Mohammed et al. 2020; Shang et al. 2021) or the studies are only available in Chinese (Gao et al. 2021; Li et al. 2021; Qiu et al. 2016).

Hałatek et al. (2005, 2008) examined 50 workers in an aluminium smelting plant who had average concentrations of 43.6 µg aluminium/l urine (95% CI: 37.5–50.2) and 42 control subjects for changes in neurophysiological parameters. The studies were not used for evaluation as the parameters used did not meet the standard.

Workplace studies of employees in the aluminium industry without measurements of aluminium in the air but with determination of the concentrations in plasma and serum can be found in Table 1.

**Tab. 1:** Neurotoxicity investigations of employees in the aluminium industry without aluminium measurements in the air

**Collective,^a)^** **country**	**Al concentration serum/plasma** **[µg/l]**	**Results**	**Performance**	**References**
**Cross-sectional studies**				
aluminium processing, 831 ♂ Al exposed in total, age: group 1: 207 (40.25 ± 8.32 years) group 2: 208 (41.52 ± 7.87 years) group 3: 208 (40.46 ± 7.96 years) group 4: 208 (39.66 ± 7.33 years) years of exposure: group 1: 16.37 ± 9.87 group 2: 17.36 ± 9.61 group 3: 15.84 ± 10.31 group 4: 14.85 ± 8.63, China workers wore protective clothing, masks, goggles, face masks and gloves; Al concentration in drinking water < WHO guideline value (200 µg/l)	plasma: mean: 15.26 group 1: < 8.28 group 2: 8.28–15.26 group 3: 15.26–27.02 group 4: > 27.02	mean values, comparison group 1 and 4: stat. sig. in the trend test** and in partial correlation analysis*: total CDT score ↓, visuospatial and executive functions ↓, adjusted for age, education, income, marital status, workplace, drinking/smoking habits	**questionnaire:** age, gender, level of education and income, marital status, lifestyle (smoking status, drinking habits), work history, medical history and use of medication, family medical history **exclusion criteria:** diseases that cause cognitive impairment, family history of dementia, regular use of medication (containing Al or affecting CNS), vision/hearing problems **cognitive function tests:** MMSE, CDT, multi domain cognition	Wang et al. 2020
aluminium processing, 172 ♂ Al exposed (age: 40.89 ± 5.77 years), years of exposure: 6.64 ± 6.37 245 controls (age: 41.63 ± 5.38 years), China workers wore work clothes, masks and gloves; Al concentration in drinking water < national standard for drinking water (< 200 µg/l).	plasma (median (P25–P75)): Al exposed: 21.18 (11.84–40.54) n = 55: < 14.9 n = 117: > 14.9 controls: 10.46 (5.32–19.24) n = 153: < 14.9 n = 92: > 14.9	test results (mean ± SD): Al exposed **↓: MMSE: 27.93 ± 1.91, CDT: 2.70 ± 1.03 DS: 10.97 ± 1.96 FOME: 23.60 ± 3.12 controls: MMSE: 28.62 ± 1.25 CDT: 3.16 ± 0.86 DS: 12.24 ± 2.15 FOME: 25.80 ± 2.84 multivariate regression analysis: Al plasma level ↑: increased risk of impairment in CDT* (OR: 1.79; 95% CI: 1.13–2.84), for memory and learning** (OR: 1.88; 95% CI: 1.2–2.9), for visual-spatial and executive dysfunctions (OR: 2.02; 95% CI: 1.11–3.66); adjusted for age, level of education, income, smoking and drinking habits	**exclusion criteria:** diseases that cause cognitive impairment, any family history of dementia, regular use of medication (containing Al or affecting CNS), vision/hearing problems, missing demographic data and blood samples **cognitive function tests:** MMSE, CDT, DS, FOME	Meng et al. 2019 b
aluminium processing, 853 ♂ employees, thereof 53 cases of MCI (age: 45.04 ± 6.15 years), years of exposure: < 1 (n = 34) > 1 (n = 19) 212 controls (age: 44.71 ± 6.11 years), China workers wore work clothes, masks and gloves; Al concentration in drinking water < national standard for drinking water (< 200 µg/l)	plasma (median (P25–P75)): Al exposed with MCI: 18.17 (10.39–34.96) n = 18: < 13.13 n = 35: > 13.13 controls: 12.02 (6.35–20.86) n = 114: < 13.13 n = 98: > 13.13	multivariate logistic regression analysis: Al plasma level ↑: risk of cognitive impairment ↑ (AOR*: 2.24; 95% CI: 1.17–4.26), adjusted for education, smoking and drinking habits smoking status and alcohol consumption: no association for cognitive impairment	**exclusion criteria:** diseases causing cognitive impairment, any family history of dementia, regular use of medication (containing Al or affecting CNS), use of cookware containing Al, vision/hearing problems, missing demographic data and blood samples **cognitive function tests:** MMSE, CDT (values not presented)	Meng et al. 2019 a
aluminium production, 576 ♂ Al-exposed, thereof 85 cases of MCI (MMSE < 24 points, CDT < 3) (age: 39.86 ± 10.09 years), 85 controls (age: 40.15 ± 9.46 years) working years: Al workers with MCI: 16.91 ± 9.02 controls: 16.53 ± 8.56, Asia (no other details)	serum (mean ± SD): Al exposed with MCI: 85.18 ± 23.68 (↑**) controls: 33.13 ± 14.11	Al exposed with MCI: MMSE ↓*, CDT ↓*, adjusted for potential confounders (no other details) *PI3K/Akt/mTOR1* gene expression ↓*	**questionnaire:** demographic data (e. g. age, gender), workplace data (e. g. years worked, protective clothing), medical history, lifestyle habits (e. g. alcohol consumption, smoking) **exclusion criteria:** age > 60 years, literacy level less than primary school, duration of employment in Al production < 10 years, diagnosis of mental or neurodegenerative disease, family medical history of neurodegenerative diseases, use of psychotropic substances > 1 month, use of antacid and/or Al-containing foods or use of aluminium cookware > 1 month, severe noise pollution, aphasia or hearing loss, extremely uncoordinated or ill appearance **cognitive function tests:** MMSE, CDT	Shang et al. 2020
aluminium electrolysis, 187 ♂ Al-exposed, thereof 49 without MCI (age: 41 years (35.5–45.5)) 138 with MCI (age: 42.5 years (35–48)), Asia	plasma (median (IQR)): group without MCI: 55.86 (38.70–77.01) group with MCI: 72.79 (42.51–102.65)	total MoCA scores: group without MCI: ≥ 26 group with MCI: < 26	**questionnaire and exclusion criteria:** see Shang et al. 2020 **cognitive function tests:** MoCA	Shang et al. 2021
aluminium processing, 1660 ♂ Al exposed age [years]: younger employees (< 40 years, number not specified) group 1: 32.35 ± 4.89 group 2: 32.77 ± 5.18 group 3: 30.41 ± 5.30 group 4: 32.99 ± 4.43 older employees (> 40 years, number not specified) group 1: 45.41 ± 3.70 group 2: 45.54 ± 3.72 group 3: 45.43 ± 3.70 group 4: 44.83 ± 3.40 working years: younger employees group 1: 8.94 ± 6.27 group 2: 10.77 ± 6.52 group 3: 8.34 ± 5.69 group 4: 15.28 ± 5.64 older employees group 1: 21.10 ± 7.32 group 2: 21.16 ± 8.47 group 3: 22.22 ± 7.00 group 4: 22.47 ± 8.53, China workers wore work clothes, masks and goggles	plasma: n = 415 each group 1: < 15 group 2: 15.0–34.52 group 3: 34.52–42.25 group 4: > 42.25	multiple linear regression analysis/trend test: stat. sig.* negative correlation of DST, DSBT with plasma Al concentration in all employees and in older and younger employees, adjusted for age, educational level, marital status, alcohol consumption, smoking habits and working years; logistic regression analysis: OR of group 4 younger employees DSBT: 15.31 (95% CI: 4.18–56.06)**, DST: 3.27 (95% CI: 1.62–6.62)** older employees DSBT: 7.64 (95% CI: 3.85–15.19)**, DST: 1.7 (95% CI: 1.06–2.71)*	**questionnaire:** age, level of education, marital status, alcohol consumption, smoking habits, other socio-economic and lifestyle factors. **exclusion criteria:** missing blood samples or plasma aluminium concentration measurement, employed <1 year, regular intake of Al containing stomach medication, uncooperative, suffering from mental and/or neurological disease **cognitive function tests:** MMSE, CDT, DST, FOM, VFT, SRT, ATIME, FAS, SLO	Xu et al. 2021
aluminium smelting, 66 formerly exposed to Al (age: 62.03 ± 7.09 years), 70 controls (age: 60.77 ± 7.95 years) working years: Al exposed: 30.18 ± 7.23 controls: 31.54 ± 5.98 alcohol consumption (more than 3 times a week in the last 6 months): Al exposed: 72.7% controls: 61.4%, China	serum (mean ± SEM): formerly exposed to Al: 25.18 ± 2.65 ↑** controls: 9.97 ± 2.83	cognitive function: MMSE (adjusted for age and level of education) exposed: 26.13 ± 2.57 ↓**, controls: 27.89 ± 1.91 MCI cases: exposed: 12 (18.2%); controls: 4 (5.7%) MCI cases vs. non-MCI: stat. sig.* ↑: tau5, p-tau181, p-tau231, p-tau396 Al exposed vs. control: stat. sig.* ↑ p-tau231, p-tau181	**grouped by:** age, level of education, socio-economic status, lifestyle, health **questionnaire:** age, level of education, lifestyle (smoking status, drinking habits), work history, personal and family medical history **exclusion criteria:** regular use of medication (e. g. antacids), head trauma, kidney damage, visual/hearing problems, psychiatric, somatic, neurological disease **cognitive function test:** MMSE examination of peripheral blood lymphocytes: protein expression of t-tau (tau5), p-tau396, p-tau262, p-tau231, p-tau181	Lu et al. 2014
**Longitudinal studies**				
aluminium processing, 276 Al exposed (♂) (age: 37.9 ± 7.8 years) total, group 1: n = 91 (38.0 ± 8.2 years) group 2: n = 93 (38.8 ± 7.5 years) group 3: n = 92 (36.8 ± 7.6 years) working years: total: 11.3 ± 8.2 group 1: 12.0 ± 8.7 group 2: 12.3 ± 8.5 group 3: 9.5 ± 7.2, China	plasma (range): median (P25–P75): total: 27.69 (12.47–46.01), group 1 (< 17.6): 7.82 (4.51–12.39) group 2 (17.6–37.3): 27.58 (22.50–32.92) group 3 (> 37.3): 63.01 (46.01–93.19)	generalised linear regression: 2014 no stat. sign. association between plasma Al concentration and cognitive performance 2016–2014: plasma Al concentration negatively associated with MMSE2016–2014*, DSBT2016–2014*, FOME2016–2014**, VFT2016–2014* multivariate logistic regression: Al in plasma ↑ increases the risk of a FOME score decrease stat. sig. **, adjusted for age, level of education, marital status, drinking/smoking habits, working years, workplace	**exclusion criteria:** known mental or neurological impairment, medication intake (antacids), lack of follow-up, employed for less than 2 years, uncooperative **questionnaire:** age, gender, level of education, marital status, lifestyle habits (smoking status, drinking habits), medical history **cognitive function tests** (2014 and follow up in 2016): MMSE, CDT, ATIME, FOME, DS, DSFT, DSBT, VFT, FAS, SLO	Lu et al. 2021

* p < 0.05;

** p < 0.01; Al: aluminium; AOR: adjusted odds ratio; ATIME: average reaction time; CDT: clock drawing test; CFT: category fluency repetition; DS: digit span test; DSBT: digit span backward test; DSFT: digit span forward test; FAS: fastest reaction time; FOME: Fuld Object-Memory Evaluation; MCI: mild cognitive impairment; MMSE: mini-mental state examination; MoCA: The Montreal Cognitive Assessment; P25: 25^th^ percentile; P75: 75^th^ percentile; SD: standard deviation; SEM: standard error of the mean; SLO: slowest reaction time; SRT: simple reaction time; stat. sig.: statistically significant; VFT: verbal fluency test

^a)^Aluminium serum/plasma levels in all control and comparison groups comparatively high (reference value general population < 5 µg/l according to HBM-Kommission 1998).

## Re-evaluation of the BAT value

There is hardly any information on lung diseases caused by aluminium dust, most of which originate from the aluminium powder industry. Of the studies described that show effects on the respiratory system, none is conclusive enough to derive a limit value for lung changes. A significant correlation between the development of aluminosis and aluminium was observed at concentrations in urine of 200 µg aluminium/g creatinine and above (Kraus et al. 2006).

The MAK values for poorly soluble and soluble aluminium compounds were derived for the local effects in the lungs (Hartwig and MAK Commission 2025 a, b). The BAT value for aluminium is based on the neurotoxicity resulting from systemic internal exposure. Neurotoxic effects were considered the most sensitive systemic endpoint of aluminium and a BAT value of 50 μg aluminium/g creatinine was derived (Klotz et al. 2019) based on a NOAEL of 50 μg/g creatinine for the occurrence of preclinical neurotoxic effects in humans, which was determined using standardised neuropsychological test procedures in workplace studies. As the BAT value is thus well-founded and there are no recent data that would call this into question and could be used to derive a limit value,

**the BAT value for aluminium of 50 μg aluminium/g in urine creatinine is confirmed.**

Sampling is at the end of the shift, for long-term exposures after several previous shifts.

## Interpretation 

Aluminium compounds have very different solubilities. Therefore, MAK values for poorly soluble aluminium compounds of 0.05 mg Al/m^3^ R (0.5 mg Al/m^3^ I) and for soluble aluminium compounds of 0.005 mg Al/m^3^ I for aluminium chlorohydrate and 0.0002 mg Al/m^3^ I for aluminium chloride, aluminium citrate, aluminium lactate, aluminium nitrate and aluminium sulphate were derived. Measuring the air concentrations is often problematic. In addition, there is often a very heterogeneous mixture of aluminium compounds (e. g. during aluminium welding). In biomonitoring, the bioavailable part of soluble and poorly soluble aluminium compounds are recorded. Therefore, in the case of inhalation exposure to aluminium compounds, not only the air concentration must be measured, but also biomonitoring must be carried out to be able to reliably assess occupational exposure. On the other hand, observance of the BAT value does not release the employer from compliance with the air limit value, particularly in the case of soluble aluminium compounds.

This BAT value does not protect against irritation or local effects of soluble aluminium compounds.

In the pre-analytical phase, contamination of the samples can be largely prevented by using suitable urine beakers, which are only opened directly before the sample is taken, and by sending the urine sample in this beaker without decanting it.

## Prenatal toxicity

Developmental neurotoxicity has to be evaluated for substances whose MAK or BAT value was derived from a neurotoxic effect. The BAT value for aluminium was derived from neurotoxicity.

### Epidemiological studies

There are several environmental epidemiological studies on aluminium exposure in pregnant women (Table 2; see also Hartwig and MAK Commission 2025 a, b).

**Tab. 2:** Environmental epidemiological studies on aluminium concentrations in the urine of pregnant women

**Country**	**Collective (age: mean ± SD; range), ** **time of urine sample**	**Aluminium in urine** **[µg/l]**	**Analytics**	**Remarks**	**References**
British Columbia, Canada	31 ♀ (21–41 years), about 18.5^th^ week of pregnancy, pilot study	GM: 15.3 P10: 5.15 P95: 355.0	ICP-MS; LOD: no data; urine samples from 5 consecutive days	Al determined in 29 ♀	Caron-Beaudoin et al. 2019
Houston, Texas, USA	131 ♀ (at least 18 years), socioeconomically disadvantaged pregnant women	GM: 23.3 mean ± SD: 45.1 ± 120.7 range: 2.12–1346.64	ICP-MS; LOD: 0.66 μg/l; spot urine	Al determined in 126 ♀	Han et al. 2020
West Australia	173 ♀ (19–44 years), about 2 weeks prior to birth, non-smokers	mean: 13.1 median: 9.1 range: < 5.0–78.7 P5: < 5.0 P95: 42.7	ICP-MS; LOD: 5.0 μg/l		Callan et al. 2013
Israel	of Bedouin-Arab origin, > LOD: 15 ♀ (30.8 ± 7.1; 20–41 years), < LOD: 43 ♀ (27.6 ± 6.1; 20–42 years), on arrival at the hospital for the birth	GM: 12 range: 9–28.3	GF-AAS; LOD: 9 μg/l; spot urine	association between Al and small for gestational age	Karakis et al. 2014
Israel	of Bedouin-Arab origin, > LOD: 37 ♀ (30.8 ± 7.1; 20–41 years), < LOD: 103 ♀ (no data), on arrival at the hospital for the birth	samples > LOD: GM: 12.2 95%-CI: 10.9–12.6 range: 7.2–28.3	GF-AAS; LOD: 9 μg/l; spot urine	association between Al and minor anomalies	Karakis et al. 2015
Israel	of Bedouin-Arab origin, 141 ♀ (28.1 ± 6.1 years), on arrival at the hospital for the birth	GM: 6.95 μg/l 95%-CI: 4.63–10.43 maximum: 412.43	ICP-MS; LOD: 0.01 μg/l; spot urine		Karakis et al. 2020
Israel	of Bedouin-Arab origin, 110 ♀ (28.1 ± 6.3; 18.4–41.7 years), on arrival at the hospital for the birth	GM: 6.14 95%-CI: 3.80–9.90) range: 0.01–97.27	ICP-MS; LOQ: 0.01 μg/l; spot urine	association between Al and premature birth or the occurrence of malformations	Karakis et al. 2021
South Africa	450 ♀ (24.8 ± 6.2; 14–49 years), on arrival at the hospital for the birth	GM: 13.1 95%-CI: 11.97–14.35 μg/l mean ± SD: 18.1 ± 14.9 range: 2.2–106.3	ICP-MS; LOD: 1.71 μg/l	Al determined in 318 ♀	Röllin et al. 2018
French Guyana	geophagy group (long-term nutrition with clay; Hb ≤ 85 g/l): 98 ♀ (26 ± 7.3 years), control group (Hb > 105 g/l): 85 ♀ (27 ± 6.4 years), about 2^nd^ trimester	geophagy group: mean ± SD: 92.8 ± 251.2; control group: mean ± SD: 12.1 ± 23	ICP-MS; LOD: no data	pemba (clay product for nutrition) as a source of Al	Lambert et al. 2010
Mexico City, Mexico	188 ♀ (age not specified), 3^rd^ trimester	AM: 37.6; GM: 25.3 median: 24.6 μg/l IQR: 14.6; 43.9 maximum: 333	ICP-MS; LOQ: 8.6 μg/l		Lewis et al. 2018
Wuhan, China	746 ♀ (28.6 ± 3.3 years) in the 1^st ^trimester (13^th^ week of pregnancy), thereof 745 ♀, in the 2^nd^ trimester (24^th^ week of pregnancy) and 599 ♀ in the 3^rd^ trimester (35^th^ week of pregnancy)	1^st^ trimester: GM: 23.6 95%-CI: 21.4–26.0, 2^nd^ trimester: GM: 20.4 95%-CI: 18.0–23.2, 3^rd^ trimester: GM: 28.4 95%-CI: 25.0–32.7	ICP-MS; LOD: 1.06 μg/l; spot urine		Liu et al. 2019

AAS: atomic absorption spectroscopy; AM: arithmetic mean; CI: confidence interval; GF: graphite furnace; GM: geometric mean; ICP-MS: inductively coupled plasma mass spectrometry; IQR: interquartile range; LOD: limit of detection; LOQ: limit of quantitation; P5: 5 percentile; P10: 10^th^ percentile; P95: 95^th^ percentile; SD: standard deviation

There are no reliable studies available to assess the prenatal and developmental toxicity of aluminium compounds in humans.

In children and adults, the use of aluminium-contaminated dialysates or aluminium-containing phosphate binders (for uraemia) resulted in considerable aluminium exposure. Typically, the aluminium concentrations in plasma were 100 to 200 µg/l; in severe cases over 500 µg/l. In some patients, this caused an aluminium-induced neurotoxic syndrome, also known as ‘dialysis dementia’ (ATSDR 2008). There is no evidence from the available studies that children are more sensitive to aluminium-induced neurotoxic effects than adults. These studies are not suitable for assessing the toxicity of aluminium at the workplace.

Aluminium concentrations in the urine of pregnant animals were not determined. The data on the aluminium concentration in the blood of pregnant animals do not allow any statement to be made as to the blood concentration at which developmental (neuro)toxic effects occur.

The placental transfer of aluminium from the dam to the foetus has been shown for rats and mice. The amount of aluminium that reaches the foetus depends on the concentration in maternal blood. A quantitative estimate is not possible due to a lack of data after administration over the entire gestation period.

There are major differences in the toxicokinetics of aluminium compounds between rodents and humans (see Hethey et al. 2021). Therefore, there are major uncertainties with regard to the toxicokinetic conversion.

**A reliable risk assessment for developmental toxicity and developmental neurotoxicity is not possible for the BAT value of 50 µg aluminium/g creatinine.**

The arguments for assigning the BAT value of aluminium to the new Pregnancy Risk Group B (suspected) were discussed critically by the Commission. A suspicion of Pregnancy Risk Group B could not be reliably substantiated on the basis of the available data.

The following arguments speak in favour of assigning the BAT value for aluminium to Pregnancy Risk Group D:

1. variability of aluminium exposure due to lifestyle, ubiquitous occurrence of aluminium
2. variability of aluminium concentrations in the urine of pregnant women, no statement on developmental neurotoxicity possible
3. no evidence that children are more sensitive to aluminium-induced neurotoxicity than adults
4. the amount of aluminium that reaches the foetus is presumably dependent on the concentration in the maternal blood (EFSA 2008). With a half-life of 5 hours, no significant accumulation in the blood is to be expected.
5. aluminium concentrations in the urine of pregnant animals not determined; blood concentrations causing developmental toxicity/developmental neurotoxicity in animals are unknown
6. uncertainties in the transfer of animal data to humans

Therefore, **aluminium is assigned to Pregnancy Risk Group D at a BAT value of 50 μg aluminium/g creatinine.**
